# The role of radical prostatectomy for the treatment of metastatic prostate cancer: a systematic review and meta-analysis

**DOI:** 10.1042/BSR20171379

**Published:** 2018-01-17

**Authors:** Yi Wang, Zhiqiang Qin, Yamin Wang, Chen Chen, Yichun Wang, Xianghu Meng, Ninghong Song

**Affiliations:** Department of Urology, First Affiliated Hospital of Nanjing Medical University, Nanjing, Jiangsu Province, China

**Keywords:** cytoreductive prostatectomy, metastatic prostate cancer, meta-analysis, radical prostatectomy

## Abstract

The recommended therapy by EAU guidelines for metastatic prostate cancer (mPCa) is androgen deprivation therapy (ADT) with or without chemotherapy. The role of radical prostatectomy (RP) in the treatment of mPCa is still controversial. Hence, a meta-analysis was conducted by comprehensively searching the databases PubMed, EMBASE and Web of Science for the relevant studies published before September 1st, 2017. Our results successfully shed light on the relationship that RP for mPCa was associated with decreased cancer-specific mortality (CSM) (pooled HR = 0.41, 95%CI = 0.36–0.47) and enhanced overall survival (OS) (pooled HR = 0.49, 95%CI = 0.44–0.55). Subsequent stratified analysis demonstrated that no matter how RP compared with no local therapy (NLT) or radiation therapy (RT), it was linked to a lower CSM (pooled HR = 0.36, 95%CI = 0.30–0.43 and pooled HR = 0.56, 95%CI 0.43–0.73, respectively) and a higher OS (pooled HR = 0.49, 95%CI = 0.44–0.56 and pooled HR = 0.46, 95%CI 0.33–0.65, separately). When comparing different levels of Gleason score, M-stage or N-stage, our results indicated that high level of Gleason score, M-stage or N-stage was associated with increased CSM. In summary, the outcomes of the present meta-analysis demonstrated that RP for mPCa was correlated with decreased CSM and enhanced OS in eligible patients of involved studies. In addition, patients with less aggressive tumors and good general health seemed to benefit the most. Moreover, no matter compared with NLT or RT, RP showed significant superiority in OS or CSM. Upcoming prospective randomized controlled trials were warranted to provide more high-quality data.

## Introduction

Prostate cancer (PCa) was the most common solid tumor diagnosed among the male population, with 180890 newly estimated cases and 26120 newly estimated deaths in USA, 2016 [1]. Although most of the PCa followed an indolent course with an estimated 5-year survival rate of 98.9% as well as the widely usage of prostate-specific antigen (PSA) plus digital rectal examination (DRE) in screening, it still ranked second leading cause of mortality in the western countries [2]. Generally, the mainstay of therapy for men with clinically localized PCa was surgery or radiation, and good outcomes had been verified [3]. But for metastatic prostate cancer (mPCa), as a consequence of surgical complications which could be life threatening as well as the poor oncological outcome of tumor invasion into the rhabdosphincter, rectal wall or seminal vesicles, radical prostatectomy (RP) was not recommended [4]. The recommended therapy by EAU guidelines for mPCa was androgen deprivation therapy (ADT) with or without chemotherapy [5].

The ‘premetastatic niche’ theory put forward by Kaplan et al. demonstrated that the primary tumor could act as the predominant source of metastasis through circulating tumor cells [6]. The following mouse models by Cifuentes et al. verified the theory that by removal of the primary tumor, the development of new metastasis could be prevented [7]. Meanwhile, several studies had successfully confirmed the benefit of cytoreductive surgery for other metastatic malignancy such as renal and ovarian cancers [8,9] and in which two aspects of the role highlighted, reducing the overall tumor burden and interrupting the re-seeding of the primary tumor [10,11]. As a result, selected patients could benefit from a lower risk of local complications, prolonged survival and reduced mortality [11,12]. However, in spite of the enthusiasm of cytoreductive surgery, whether it was equally feasible or beneficial in mPCa patients still remained controversial and the merits of such an approach should be carefully considered [13–16].

Over the past two decades, significant developments had been achieved in chemotherapy and androgen axis therapies. However, disappointing was no parallel increase had been seen in the overall survival (OS) or cancer-specific survival (CSS) among mPCa patients [17]. Thanks to the progresses in robotic-assisted radical prostatectomy (RP) and radiation therapy (RT) techniques, making the treatment of localized PCa be more safer and more effective, and paving the way for the treatment of mPCa [18]. Recently, by utilizing the aforementioned methods, Moschini et al. had successfully demonstrated the feasibility of local surgical treatment of the primary tumor in mPCa patients. However, no survival benefits had been observed. Meanwhile, Leyh-Bannurah et al. shed light on that RP or RT could result in a lower mortality compared with no local therapy (NLT). Thus, the opinion of RP in the treatment of mPCa remained inconsistent.

In summary, along with the successful application of cytoreductive surgery for other metastatic malignancy and the progresses in surgical techniques, the role of cytoreductive RP for mPCa had gained a lot of interest. However, researches had not reached a consensus. Hence, a meta-analysis was conducted to shed light on the merits of such an approach by cancer-specific mortality (CSM) or OS based on available data.

## Materials and methods

### Search strategy

We conducted a comprehensive search of the databases PubMed, EMBASE and Web of Science to identify relevant literature up to September 1st, 2017. The search strategy was consisted of the following keywords in combination with Medical Subject Headings (MeSH) terms and text words: ‘mPCa’ or ‘metastatic prostate neoplasms’ or ‘metastatic cancer of the Prostate’ or ‘metastatic neoplasms of the Prostate’ or ‘mPCa’ or ‘radical prostatectomy’ or ‘RP’ or ‘cytoreductive prostatectomy’. The major inclusion criteria were as follows: (1) English studies; (2) patients with mPCa; (3) focused on the relationship of radical prostatectomy for mPCa; (4) sufficient data could be extracted. The major exclusion criteria were as follows: (1) non-English research; (2) duplicates of the previous publication; (3) reviews or letters or case reports or comments or editorials; (4) unrelated to mPCa or RP; (5) absence of key information.

### Data extraction

All eligible researches were independently determined by two blind reviewers (Y.W. and Z.Q.Q), based on the inclusion and exclusion criterion. Disagreements were addressed by consultation with a third reviewer (Y.M.W.). The following information was extracted from included articles: first author's name, publication year, median or mean age, dominant ethnicity, study design, survival analysis, source of hazard ratio (HR), months of follow-up, number of patients, treatment, HR and 95% confidence interval (CI), Gleason Score, most PSA, M-stage, N-stage. Data were extracted from Kaplan–Meier curves to extrapolate HRs with 95% CIs using previously described methods, if it could not be directly obtained from each article [19,20].

### Quality assessment

Two blind reviewers independently performed the methodological quality assessment of eligible studies according to the Newcastle–Ottawa Scale (NOS) (http://www.ohri.ca/programs/clinical_epidemiology/oxford.htm), which was one of the most useful scale to evaluate the quality of non-randomized studies [21]. The criteria of quality assessment were as follows: (1) representativeness of the exposed cohort; (2) selection of the non-exposed cohort; (3) ascertainment of exposure; (4) outcome of interest not present at start of study; (5) control for important factor or additional factor; (6) assessment of outcome; (7) follow-up long enough for outcomes to occur; (8) adequacy of follow up of cohorts. Each quality choice could be awarded a maximum of one star except for the numbered 5 item which could be granted a maximum of two stars. Total quality scores ranged from 0 to 9. If the final score >6, we regarded it as high quality. Detailed rankings for each study were shown in Table 1.

**Table 1:** Newcastle–Ottawa Quality Assessments Scale

Studies	Year	Quality indicators from Newcastle–Ottawa Scale								Scores
		1	2	3	4	5	6	7	8	
Parikh	2017	★	★	−	★	★★	★	★	★	8
Moschini	2017	−	★	★	★	★★	−	★	★	7
Leyh-Bannurah	2017	★	−	★	★	★★	★	★	★	8
Rusthoven	2016	★	★	★	★	★★	★	−	−	7
Satkunasivam	2015	★	★	★	★	★★	−	−	−	6
Culp	2014	★	★	−	★	★★	−	★	−	6
Antwi	2014	★	★	★	★	★★	−	−	−	6
Shao	2014	★	★	★	★	★★	−	★	★	8
Gratzke	2014	★	★	★	★	★★	−	−	−	6

1. Representativeness of the exposed cohort; 2. selection of the non-exposed cohort; 3. ascertainment of exposure; 4. outcome of interest not present at start of study; 5. control for important factor or additional factor; 6. assessment of outcome; 7. follow-up long enough for outcomes to occur; 8. adequacy of follow up of cohorts.

### Statistical analysis

The relationship between RP and mPCa was conducted by CSM or OS based on available data and the pooled HRs with 95% confidence intervals (CIs) were utilized to evaluate its efficacy. The Chi-square test and I-square test were utilized to assess the heterogeneity. If the Chi-square test *P*<0.1 or *I*^2^>50%, it was considered to be significant heterogeneity. According to the presence or absence of significant heterogeneity (*P*<0.1 or *I*^2^>50%), the random-effects model (DerSimonian–Laird method) or the fixed-effects model (Mantel–Haenszel method) was applied respectively [22]. Moreover, in the case of significant heterogeneity, subgroup analysis was carried out by treatment, different levels of Gleason score, M-stage or N-stage to further minimize the influence. Sensitivity analysis was conducted to access the stability of results by deleting one single study each time to reflect the impact of the individual to overall. Furthermore, Begg's funnel plot was conducted for potential publication bias and Egger's test was performed to assess funnel plot asymmetry statistically and if *P*<0.05, it indicated the existence of publication bias [23]. Besides, *P* values were adopted by a two-sided test and *P*<0.05 was considered to be statistically significant. In addition, all statistical data were conducted by Stata software (version 12.0; StataCorp LP, College Station, TX).

## Results

### Characteristics of enrolled studies

**Figure 1:**
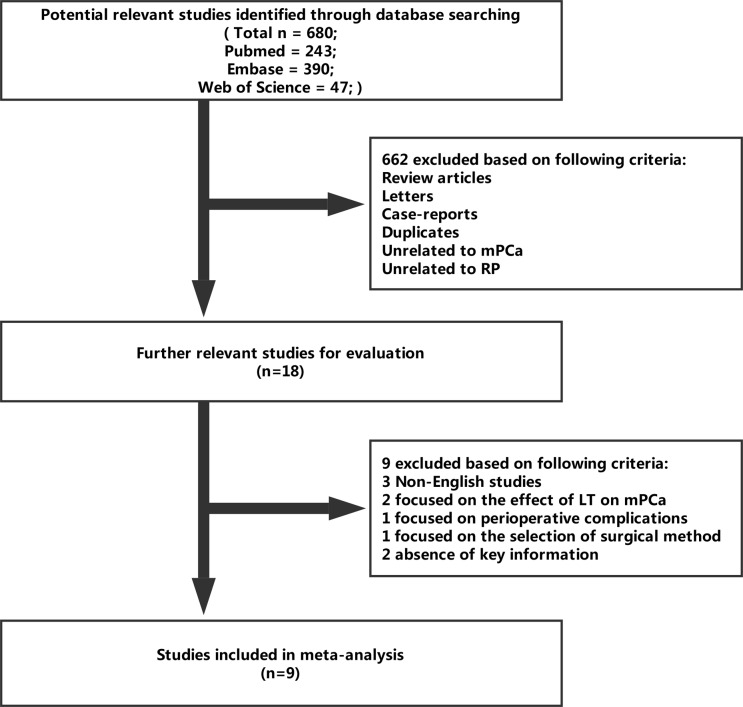
Flow diagram of the literature selection process.

A total of 680 relevant studies were comprehensively identified through databases and enrolled in the present meta-analysis by previous search strategy. A total of 662 records were excluded because of review articles, letters, case-reports, duplicates, unrelated to mPCa or unrelated to RP after screening the tittles and abstracts. Of the remaining 18 studies, 3 studies were non-English studies; 2 studies focused on the effect of local therapy (LT) on mPCa; 1 study focused on perioperative complications; 1 study focused on the selection of surgical method; 2 studies were lack of key information (HRs or survival curves). Finally, nine studies were eligible for this study [24–32] (Figure 1).

The detailed characteristics of these nine enrolled studies with 36947 participants were summarized in Table 2. They were all retrospective cohort study. Of the nine included studies, three articles focused on the OS and the remaining six focused on the CSM. When taking the treatment item into account, most of the studies focused on RP vs NLT, except for Leyh-Bannurah et al and Gratzke et al. who concentrated on not only RP vs NLT, but also RP vs RT [24,27]; and Shao et al. who merely concentrated on RP vs RT. Amongst them, Shao et al. even divided RP vs RT into a low risk and an intermediate-high-risk group based on Gleason score and cancer stage [26]. Data were directly obtained from articles except for the study by Gratzke et al. whose data were extracted from Kaplan–Meier curves to extrapolate HRs with 95% CIs [27]. The pooled HRs and 95% CIs of different levels of Gleason score, M-stage and N-stage were detailed in Table 3. Owing to the scantiness of data, merely the data of CSM was analyzed and the data of OS was omitted.

**Table 2:** Main characteristics of individual studies included in the meta-analysis

First author	Publication year	Median or mean age	Dominant ethnicity	Study design	Survival analysis	Source of HR	Months of follow-up	Number of patients	Treatment	HR (95% CI)	Most Gleason score	Most PSA
**Parikh**	2017	72	Caucasion	R	OS^M^	Reported	22 months median	6051	RP vs NLT	0.51 (0.45–0.59)	8–10	>20
**Moschini**	2017	61	NA	R	CSM^U^	Reported	38.8 months median	47	RP vs NLT	0.53 (0.17–1.69)	7–10	>20
**Leyh-Bannurah**^1^	2017	64	Caucasion	R	CSM^M^	Reported	NA	2370	RP vs NLT	0.35 (0.26–0.46)	7–10	>20
**Leyh-Bannurah**^2^	2017	64	Caucasion	R	CSM^M^	Reported	NA	2370	RP vs RT	0.59 (0.35–0.99)	7–10	>20
**Rusthoven**	2016	69	Caucasion	R	OS^M^	Reported	120 months maximum	5913	RP vs NLT	0.38 (0.25–0.58)	7–9	>20
**Satkunasivam**	2015	73	African American	R	CSM^M^	Reported	NA	4069	RP vs NLT	0.58 (0.35–0.95)	7–8	>30
**Culp**	2014	62	Caucasion	R	CSM^M^	Reported	27 months median	8185	RP vs NLT	0.37 (0.26–0.54)	7–10	>20
**Antwi**	2014	65	Caucasion	R	CSM^M^	Reported	80 months maximum	7858	RP vs NLT	0.28 (0.20–0.39)	7–10	>10
**Shao**^3^	2014	75	Caucasion	R	CSM^M^	Reported	33 months median	916	RP vs RT	0.68 (0.38–1.22)	5–7	NA
**Shao**^4^	2014	75	Caucasion	R	CSM^M^	Reported	33 months median	916	RP vs RT	0.51 (0.36–0.73)	5–7	NA
**Gratzke**^1^	2014	NA	NA	R	OS^M^	SC	120 months maximum	1538	RP vs NLT	0.48 (0.35–0.68)	NA	>20
**Gratzke**^2^	2014	NA	NA	R	OS^M^	SC	120 months maximum	1538	RP vs RT	0.46 (0.33–0.65)	NA	>20

NA: not available; R: retrospective study; OS: overall survival; CSM: cancer-specific mortality; U: univariate analysis; M: multivariate analysis; SC: survival curves. ^1^The treatment group of RP vs NLT. ^2^The treatment group of RP vs RT. ^3^The low risk group. ^4^The intermediate-high risk group.

**Table 3:** HRs and 95%CIs of different levels of Gleason score, M-stage and N-stage in enrolled studies

First author	Year	Study design	Survival analysis	Source of HR	Gleason score	HR (95% CI)	M-stage	HR (95% CI)	N-stage	HR (95% CI)
**Moschini**	2017	R	CSM^U^	NA	NA	NA	NA	NA	NA	NA
**Leyh-Bannurah**^1^	2017	R	CSM^M^	Reported	≥8 vs ≤7	1.84 (1.59–2.13)	M1c vs M1a	1.98 (1.52–2.58)	N1 vs N0	1.18 (0.91–1.52)
**Leyh-Bannurah**^2^	2017	R	CSM^M^	Reported	≥8 vs ≤7	3.67 (2.03–6.66)	M1c vs M1a	4.7 (1.88–11.7)	N1 vs N0	1.01 (0.34–2.99)
**Satkunasivam**	2015	R	CSM^M^	Reported	≥7 vs ≤6	1.66 (1.32–2.1)	M1c vs M1a	1.93 (1.49–2.51)	N1 vs N0	1.13 (0.98–1.29)
**Culp**	2014	R	CSM^M^	Reported	≥8 vs ≤7	1.7 (1.42–2.04)	M1c vs M1a	2.35 (1.94–2.85)	N1 vs N0	1.21 (1.09–1.33)
**Antwi**	2014	R	CSM^M^	Reported	≥7 vs ≤6	1.71 (1.44–2.04)	M1c vs M1a	2.19 (1.83–2.63)	NA	NA
**Shao**^3^	2014	R	CSM^M^	NA	NA	NA	NA	NA	NA	NA
**Shao**^4^	2014	R	CSM^M^	NA	NA	NA	NA	NA	NA	NA
**Parikh**	2017	R	OS^M^	NA	NA	NA	NA	NA	NA	NA
**Rusthoven**	2016	R	OS^M^	Reported	NA	NA	NA	NA	N1 vs N0	1.053 (0.973–1.140)
**Gratzke**^1^	2014	R	OS^M^	NA	NA	NA	NA	NA	NA	NA
**Gratzke**^2^	2014	R	OS^M^	NA	NA	NA	NA	NA	NA	NA

NA: not available; R: retrospective study; U: univariate analysis; M: multivariate analysis; OS: overall survival; CSM: cancer-specific mortality. ^1^The treatment group of RP vs NLT. ^2^The treatment group of RP vs RT. ^3^The low risk group. ^4^The Intermediate-high risk group.

### CSM associated with mPCa

**Figure 2:**
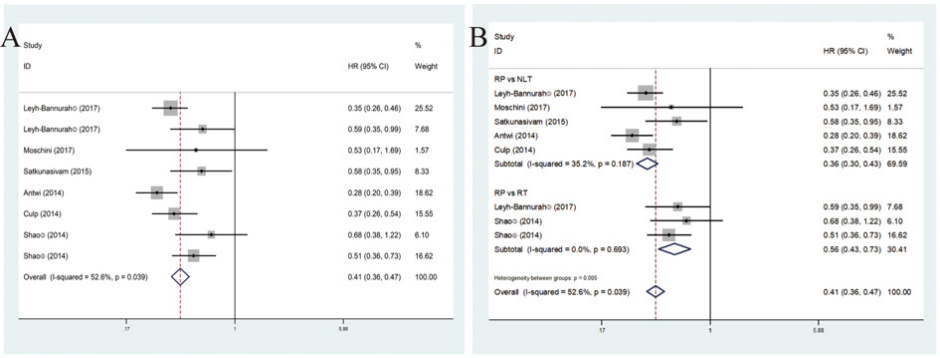
Forest plots of CSM in association with RP for mPCa. (**A**) The overall CSM; (**B**) the subgroup analysis of CSM.

These six included studies revealed a prognostic role of RP for mPCa on CSM by fixed-effects model based on moderate heterogeneity (*P* = 0.039, *I*^2^ = 52.6%). RP for mPCa was linked to decreased CSM (pooled HR = 0.41, 95%CI = 0.36–0.47) (Figure 2A). In subsequent stratified analysis according to treatment, the heterogeneity was further reduced and RP vs NLT was linked to lower CSM (pooled HR = 0.36, 95%CI = 0.30–0.43) in the fixed-effect model (*P* = 0.187, *I*^2^ = 35.2%). Meanwhile, when compared with RT, RP showed superiority in decreasing CSM (pooled HR = 0.56, 95%CI = 0.43–0.73) in the fixed-effect model (*P* = 0.693, *I*^2^ = 0.0%) (Figure 2B).

### OS associated with mPCa

**Figure 3:**
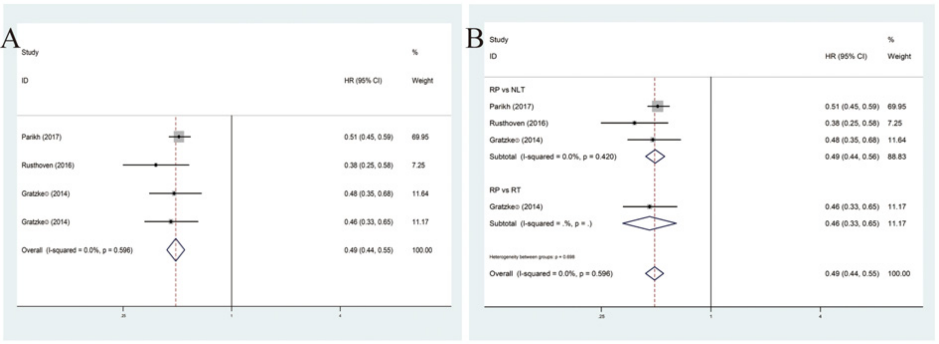
Forest plots of OS in association with RP for mPCa. (**A**) The overall OS; (**B**) the subgroup analysis of OS.

These enrolled four studies showed a positive role of RP for mPCa on OS by fixed-effects model based on the low heterogeneity among studies (*P* = 0.596, *I*^2^ = 0.0%). Our results successfully demonstrated that enhanced OS was correlated with RP for mPCa (pooled HR = 0.49, 95%CI = 0.44–0.55) (Figure 3A). Subsequent stratified analysis shed light on that no matter how RP compared NLT or RT, it was associated with an enhanced OS (pooled HR = 0.49, 95%CI = 0.44–0.56; pooled HR = 0.46, 95%CI = 0.33–0.65) (Figure 3B).

### Different levels of Gleason score, M-stage or N-stage

**Figure 4:**
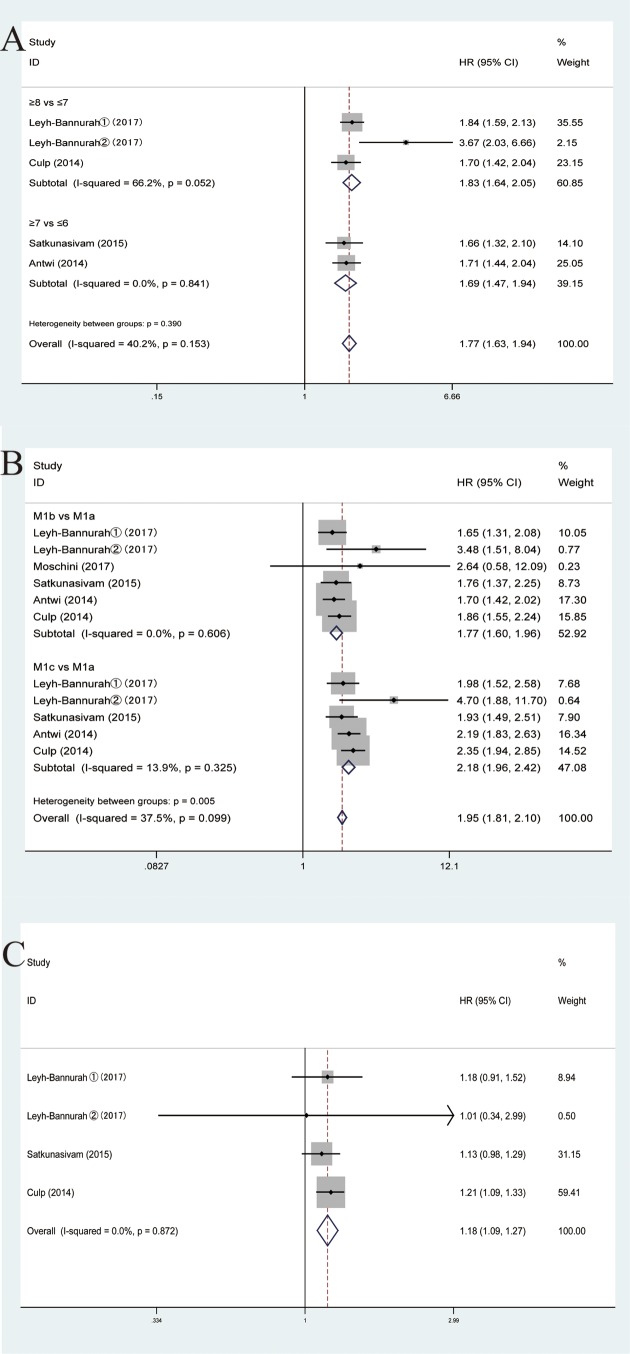
Forest plots of comparing different levels of Gleason score, M-stage or N-stage in enrolled studies. (**A**) Gleason score of CSM. (**B**) M-stage of CSM. (C) N-stage of CSM.

When comparing different levels of Gleason score, M-stage or N-stage, our results indicated that high level of Gleason score, M-stage or N-stage was associated with increased CSM (pooled HR = 1.77, 95%CI = 1.63–1.94; pooled HR = 1.95, 95%CI = 1.81–2.10 and pooled HR = 1.18, 95%CI 1.09–1.27, respectively). Subsequent stratified analysis by different levels of Gleason score, shed light on that no matter ≥8 vs ≤7 or ≥7 vs ≤6 higher level of Gleason score was associated with increased CSM (pooled HR = 1.83, 95%CI = 1.64–2.05; HR = 1.69, 95%CI = 1.47–1.94, separately). In the case of M-stage, M1b vs M1a or M1c vs M1a was also linked to increased CSM (pooled HR = 1.77, 95%CI = 1.60–1.96; HR = 2.18, 95%CI = 1.96–2.42, respectively). Similarly, in terms of N-stage, compared with N0 in the fixed-effects model, N1 was related to enhanced CSM (pooled HR = 1.18, 95%CI = 1.09–1.27). Our results revealed that high level of Gleason score, M-stage or N-stage was associated with increased CSM. In other words, a relatively low level could be prognostic (Figure 4).

### Sensitivity analysis

**Figure 5:**
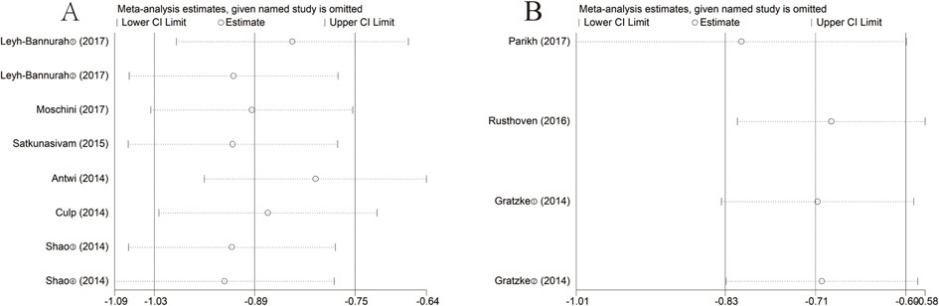
Sensitivity analysis of each included study. (**A**) CSM for individual studies. (**B**) OS for individual studies.

Sensitivity analysis was conducted to access the stability of results by deleting one single study each time to reflect the impact of the individual to overall. In the OS or CSM groups, the results did not alter significantly in the sensitivity analysis, suggesting that no individual study significantly influenced the pooled HR or the 95%CI. Namely, our results were robust (Figure 5).

### Publication bias

**Figure 6:**
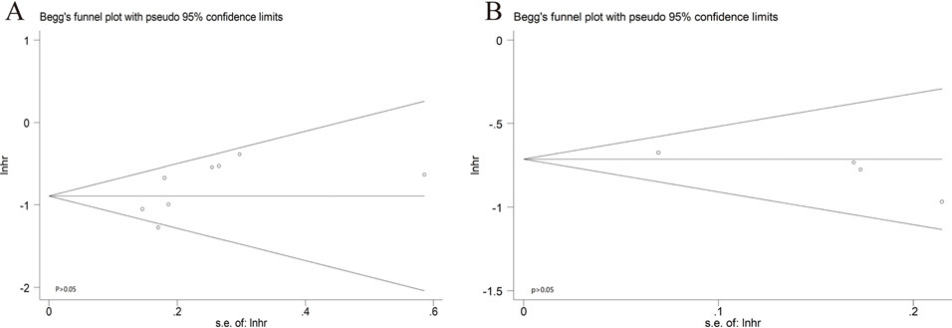
Begg's funnel plots of the publication bias. (**A**) CSM for individual studies. (**B**) OS for individual studies.

The combined application of Begg's and Egger's test was used to evaluate the publication bias and the funnel plots were displayed in Figure 6. In the pooled analysis of OS, the *P* value of Begg's test was 0.089 and the *P* value of Egger's test was 0.124. In the pooled analysis of CSM, the *P* value of Begg's test and Egger's test was 0.063 and 0.100, respectively. All of the *P* values of Begg's or Egger's test were above 0.05. In other words, there was no significant publication bias.

## Discussion

Traditionally for mPCa, ADT with or without chemotherapy was recommended by EAU guidelines. But no sign of the survival benefit had been demonstrated. Owing to the successful application of cytoreductive surgery for other metastatic malignancy accompanied by the progresses in robotic-assisted RP and RT techniques, surgical approach had shifted from low-risk disease to more advanced even high-risk tumors. Besides, it was considered to be another beneficial way. Patients who suffered from mPCa could probably benefit from the following potential sides, decreased the tumor burden, improved response to secondary treatment, immune modulation, interrupting the re-seeding of the primary tumor [33]. Interest in the role of RP for mPCa was rising. However, there was still a controversy on it. Hence, meta-analysis, as a powerful tool in providing more reliable conclusions than a single study, was carried out to clarify the merits of such an approach [34].

Our study was the first meta-analysis to shed light on the association that RP for mPCa was correlated with enhanced OS and decreased CSM in selected cases. When stratifying the researches based on the treatment, either compared with NLT (ADT or observation) or compared with RT, RP showed superiority in cutting down CSM and improving OS. When comparing different levels of Gleason score, M-stage or N-stage, our results revealed that high level of Gleason score, M-stage or N-stage was associated with increased CSM. In other words, a relatively low level of Gleason score, M-stage or N-stage could be prognostic. Subsequently sensitivity analysis and publication bias manifested the robustness of our study.

Obviously, our study had successfully demonstrated the feasibility of surgery and the survival benefit of decreasing CSM and improving OS. However, Moschini et al. concluded that compared with patients treated with ADT only, no survival benefits had been observed for patients treated with surgery in the short term. This might attribute to two aspects of reasons, the small quantity of included cases (47 cases) and the short period time of follow-up.

In selecting the appropriate surgical patient population, Fossati et al. demonstrated that the potential benefit of LT (either RP or RT) to the primary tumor among mPCa patients depended greatly on baseline characteristics and patient selection [35]. Loppenberg et al. further clarified that patients with less aggressive tumors and good general health appeared to benefit the most [36]. Associated with our results, we found that a relatively low level of Gleason score, M-stage or N-stage could be prognostic. As a result, in choosing the right surgical patients for surgery, aforementioned criteria should be taken into consideration to ensure the maximum survival benefit.

Although the feasibility and the survival benefit of surgery had been confirmed, the risk of surgery was seldom involved. Even though Sooriakumaran et al. had showed that the perioperative and short-term complication rate of RP for mPCa was not more frequent than it performed for standard indications. Owing to the lack of sufficient data, the evidence was not strong enough [37]. Meanwhile, due to the shortage of information regarding on the complications of perioperative or postoperative outcomes as well as the role of open vs robotic-assisted vs laparoscopic surgical methods for RP, we had difficulty in clarifying the risk of RP surgery based on the different periods of outcomes and the different surgical methods. Thus, more above-mentioned information was required in subsequent researches.

To some extent, several limitations should be taken into account before fully understanding this article. First, despite overall quantities of patients were huge, the number of included studies was relatively small, which brought some extent of difficulties in stratifying group. Especially in the OS group, the subsequent stratified group of Gleason score, M-stage or N-stage was omitted because of insufficient quantity of articles. Secondly, a relatively high heterogeneity in the total CSM group which caused by different ethnicity, treatment, Gleason score, PSA, T-stage, N-stage, M-stage could be reduced by subgroup analysis. In fact, merely the subgroup of treatment was carried out. The remaining was dead in the water, owing to insufficient data or the overlapping of different levels of data just like the PSA subgroup in Table 2. Thirdly, all patients were mPCa and their clinical staging varied. Due to lack of original data, further research was not carried to assess their difference. In addition, the dominant ethnicity was Caucasion or African American; but data from Asia were poorly little. Therefore, this might result into some bias. Last but not least, the included nine researches were all cohort studies derived from retrospective, observational data which could not have a clear impact on group baseline features as RCTs. Moreover, the retrospective cohort study and RCTs had a different level of evidence, which could not provide the same statistical power. Upcoming prospective RCTs were required to provide more available data. Subsequent researches should resolve the aforementioned difficulties before RP was widely used for mPCa in clinical practice.

All in all, we had successfully demonstrated the feasibility and the survival benefit of RP for mPCa in the current meta-analysis, and the potentially beneficial surgical patient was the population with a lower level of tumors and a better general health. Due to the lack of sufficient data, the risk of surgery was seldom involved. Hence, upcoming prospective RCTs were warranted to provide more available data to elaborate the efficacy.

## Conclusions

In summary, the outcomes of the present meta-analysis had shed light on the association that RP for mPCa was correlated with decreased CSM and enhanced OS in eligible patients. In addition, the patient population with a lower level of tumors and a better general health appeared to benefit the most. Meanwhile, no matter how RP compared with NLT or RT, it showed significant superiority in OS or CSM. Based on our results, we had successfully demonstrated the feasibility and the survival benefit of RP for mPCa. However, the risk of RP surgery was rarely involved, especially in the different periods of surgical complications or the different surgical methods. Subsequent prospective RCTs were needed to provide more high-quality data to elaborate the efficacy.

## Abbreviations

ADT: androgen deprivation therapy
CSM: cancer-specific mortality
mPCa: metastatic prostate cancer
NLT: no local therapy
OS: overall survival
PCa: prostate cancer
PSA: prostate-specific antigen
RP: radical prostatectomy
RT: radiation therapy
